# The Impact of Plasticizer and Degree of Hydrolysis on Free Volume of Poly(vinyl alcohol) Films

**DOI:** 10.3390/polym10091036

**Published:** 2018-09-18

**Authors:** Rebecca J. Fong, Alexander Robertson, Peter E. Mallon, Richard L. Thompson

**Affiliations:** 1 Department of Chemistry, Durham University, Mountjoy Site, Durham DH1 3LE, UK; alexander.j.robertson@durham.ac.uk (A.R.); r.l.thompson@durham.ac.uk (R.L.T.); 2 Department of Chemistry and Polymer Science, University of Stellenbosch, Matieland 7602, Western Cape, South Africa; pemallon@sun.ac.za

**Keywords:** PALS, free volume, plasticizers

## Abstract

The effect of plasticizer species and the degree of hydrolysis (DH) on the free volume properties of poly(vinyl alcohol) (PVA) were studied using positron annihilation lifetime spectroscopy. Both glycerol and propylene glycol caused an increase in the free volume cavity radius, although exhibited distinct plasticization behavior, with glycerol capable of occupying existing free volume cavities in the PVA to some extent. The influence of water, normally present in PVA film under atmospheric conditions, was also isolated. Water added significantly to the measured free volume cavity radius in both plasticized and pure PVA matrices. Differences in plasticization behavior can be attributed to the functionality of each plasticizing additive and its hydrogen bonding capability. The increase in cavity radii upon plasticizer loading shows a qualitative link between the free volume of voids and the corresponding reduction in *T*_g_ and crystallinity. Cavity radius decreases with increasing DH, due to PVA network tightening in the absence of acetate groups. This corresponds well with the higher *T*_g_ observed in the resin with the higher DH. DH was also shown to impact the plasticization of PVA with glycerol, indicating that the larger cavities—created by the weaker hydrogen bonding acetate groups—are capable of accommodating glycerol molecules with negligible effect on the cavity dimensions.

## 1. Introduction

Poly(vinyl alcohol) (PVA) is a water-soluble, synthetic, semi-crystalline polymer with excellent film forming capability, good mechanical properties, and optical transparency [1]. Its application in food packaging makes use of the excellent barrier properties of PVA to oxygen and carbon dioxide [2], but PVA is additionally valued for its solubility, nontoxicity, and biodegradability [3,4], which contribute to its low overall environmental impact. These properties, alongside its resistance to organic solvents, have led to its increased use in the laundry industry as a film for packaging unit-dose detergents.

PVA is prepared from the hydrolysis of poly(vinyl acetate) (PVAc). The characteristics of the PVA resin are therefore dependent on its degree of polymerization (DP), as well as its degree of hydrolysis (DH), which defines the fraction of hydroxyl groups present on the backbone. A lower DH (and higher proportion of residual acetate groups) leads to a lower degree of hydrogen bonding, reducing stereoregularity and decreasing the degree of crystallinity [5].

Plasticization is generally required to give PVA the properties required for many of its applications, as it is otherwise very brittle. Plasticizers are involatile, low molecular weight molecules that can modify the polymer matrix, increasing free volume and chain mobility. They are incorporated into materials in order to improve processability and flexibility, whilst maintaining the desirable mechanical properties. A variety of environmentally-benign plasticizers have been incorporated into PVA, including glycerol and propylene glycol [6,7].

The films used for laundry applications are in contact with a wide variety of detergent components which can interact with the film, and segregate to interfaces, leading to manufacturing challenges. The presence of plasticizer in PVA films, and its interaction with other molecules such as surfactants has been shown to bring about a surprisingly rich range of segregation behaviors of model film components [8,9]. The complex nature of the interactions between film components means that the prediction of migration and adsorption of different species to interfaces is difficult, even in simple models for complex formulations. Our interest is, therefore, to better understand how the composition of these films affects the properties of the polymer matrix in order to ultimately better predict and control the segregation and migration of small molecules. This understanding will also assist with the correlation of the properties of the material with film permeability and transport properties, which is particularly important in the context of the diffusion and migration of encapsulated detergent components through the film. Additionally, new theory (locally correlated lattice) and simulation (Statistical Association Fluid Theory) (SAFT) methods aimed at tackling these problems rely on detailed analysis of volume changes of mixing, which are challenging to obtain. We therefore assess positron annihilation lifetime spectroscopy (PALS) as a measure of the location of plasticizers in PVA at a molecular level, which is necessary to validate these models.

PALS can be used to probe free volume size distributions and concentrations in polymers by measuring the time difference between the emission of a positron from a radioactive source and its subsequent annihilation. It is a highly valuable technique in providing information on the polymer free volume and its relationship with various properties. These include permeability, the glass transition, crystallization, dynamics, and the chemical environment of free volume cavities. PALS has been widely used in the study of polymeric systems at a molecular level, and can be used to quantitatively assess the effect of plasticization on free volume. The plasticization of amorphous polymers has been predominantly investigated [10,11] although there have been some cases of the use of PALS to investigate the plasticization of semi-crystalline polymers, including poly(ethylene oxide) [12,13] and poly(vinyl chloride) [14,15].

The plasticization of PVA has been widely studied [6,7,16,17,18], but remarkably PALS has not been implemented to study the effect on its free volume properties. There have been a number of PALS studies of unplasticized PVA [19,20,21,22], including the incorporation of additives and the properties of nanocomposites [23,24,25,26], but most have focused on the effect of the chemical environment on the Ps yield [27,28,29]. In this work, PALS is used to assess the effect of the degree of hydrolysis and plasticizer incorporation on the free volume properties of the solution-cast PVA films, and the relationship between free volume and bulk properties such as crystallinity and glass transition temperature, *T*_g_. By exploring the plasticization of PVA and the influence of DH, insights into the polymer-additive interaction can be gained.

## 2. Materials and Methods

### 2.1. Materials

PVA, glycerol and propylene glycol were purchased from Sigma Aldrich (Gillingham, UK) and used as received. Details of the PVA resins are tabulated (Table 1).

### 2.2. Film Preparation

Solution cast films were used for all measurements. PVA was dissolved in deionised water by heating to 75 °C and stirring to create well-defined solutions at 10–20 wt % solute. Similar aqueous solutions of plasticizers were prepared. Aqueous master-batches containing the appropriate proportions of components were prepared by mixing the relevant solutions. PVA solutions with a range of DH were prepared by mixing solutions of partially- and fully-hydrolyzed resins in the appropriate proportions. Films were solution cast into aluminum dishes under atmospheric conditions (25 °C and ~50% RH) for 24 h, and subsequently dried in a vacuum oven at 25 °C for 24 h. In order to determine the influence of water content on free volume, some films were studied at intermediate stages of the drying process, or equilibrated to a defined humidity (33%) with a saturated aqueous magnesium chloride solution.

### 2.3. Positron Annihilation Lifetime Spectroscopy

PALS measurements were carried out using a fast-fast coincidence circuit. A 20 μCi ^22^Na positron source was sealed in 6 μm aluminum foil, sandwiched between two identical stacks of film (each 1–2 mm), and sealed in aluminum foil. This sandwich was then placed between the two detectors to acquire a lifetime spectrum. Each spectrum was collected to 1 million counts from annihilation events. The time resolution was monitored (to 250 ps) using a ^60^Co source. Temperature control was achieved by attaching the sample sandwich to a temperature-controlled plate placed between the two detectors. Unless otherwise specified, PALS measurements were conducted at 20 °C.

The positron decay spectra are made up of a series of lifetimes attributable to the different positron annihilation mechanisms. The lifetime data was resolved into three finite lifetime components using the PATFIT program [30]. The shortest lifetime (τ_1_) is that of *p*-Ps, the intermediate lifetime (τ_2_) is that of the “free” positron and the longest-lived component (τ_3_ > 0.5 ns) is due to the *o*-Ps pickoff lifetime. τ_3_ is correlated to the mean hole size, and was used to obtain the medium free volume cavity radius using the empirical Equation (1) [31]:

(1) $$\tau_{3}^{-1}=2\left[1-\frac{R}{R_{0}}+\frac{1}{2\pi }sin\left(\frac{2\pi R}{R_{0}}\right)\right]$$

*R* is the radius of the hole and *R*_0_ = *R +* Δ*R* where Δ*R* is the width of the electron layer at the internal surface of the potential well, determined to be 1.656 Å [32]. In the absence of positron or positronium chemical effects, the probability of *o*-Ps formation is proportional to the number of regions of low electron density (where *o*-Ps is possible). The relative intensity of the *o*-PS annihilation lifetime (*I*_3_) is a percentage of the positrons annihilating by the pickoff mechanism, and therefore related to the free volume fraction. As a result, the intensity of the *o*-Ps can be used to assess the influence of different factors on the fractional free volume change using Equation (2):

(2) $$f_{v}=CI_{3}V_{f}$$

where *f_v_* is the free volume fraction, and *V_f_* is the volume of free volume holes calculated using Equation (2) from the radius determined using Equation (1). *C* is a constant which reflects the probability of *o*-Ps formation and is independent of free volume, is estimated to be 0.0018 [32].

(3) $$V_{f}=(4\pi R^{3}/3)$$

### 2.4. Thermogravimetric Analysis

Thermogravimetric analysis (TGA) was performed on a Perkin-Elmer Pyris 1 TGA (Beaconsfield, UK) under a flow of nitrogen. Approximately 5 mg of each sample was heated at a rate of 10 °C·min^−1^ to 200 °C in order to determine the water content of the solution cast resins. As PVA degrades only above 200 °C, all mass loss in the temperature range measured was taken to be loss of residual water in the sample.

### 2.5. Dynamic Mechanical Analysis and X-ray Diffraction

Samples for XRD and DMA were prepared using the same solution casting method used to make plasticized samples for PALS. Solution cast samples were thoroughly dried under ambient conditions, then either further dried under vacuum, or equilibrated over a saturated MgCl_2_ solution at 33% relative humidity. XRD samples were prepared as 20 mm diameter disks, whereas DMA samples were 35 mm × 10 mm rectangles. In each case, samples were approximately 0.2 mm thick.

Crystallinity of PVA-30-88 and PVA-50-98 samples was examined by X-ray diffraction using Bruker D8 diffractometer (Karlsruhe, Germany) operating with a 1D detector in reflection mode using Cu Kα radiation. DMA was carried out over a temperature ramp from −40 to 100 °C at 3 °C/min with a nitrogen purge. Samples were oscillated at 1 Hz in 3-point bend mode using a TA Instruments DMA Q800 (Borehamwood, UK). The amplitude of the oscillation was up to 0.1% strain. The glass transition temperature was inferred from the maximum of the first peak in tan *δ*, where *δ* is the phase angle of the complex modulus. Because the magnitude real and imaginary components of the complex modulus are affected equally by any irregularities in sample shape, tan *δ* is independent of these variations.

## 3. Results

### 3.1. Effect of Plasticizer Inclusion of PVA Free Volume Properties

**Figure 1:**
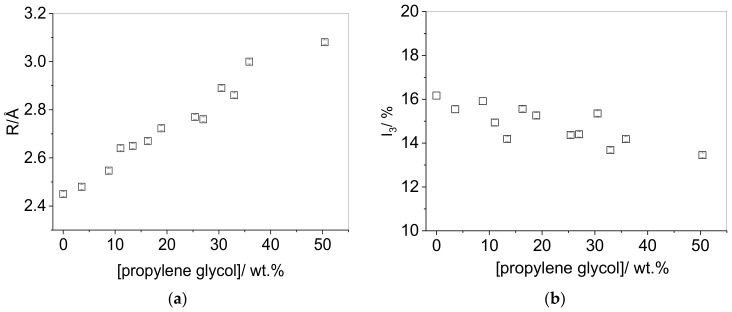
(**a**) Cavity radius (*R*) in vacuum-dried, solution cast PVA (PVA-70-88) films as a function of propylene glycol concentration, (**b**) *o*-Ps intensity (*I*_3_) in vacuum-dried solution cast PVA (PVA-70-88) films as a function of propylene glycol concentration.

All PALS results could be satisfactorily fitted using three-lifetime analysis. Two model plasticizers were studied: propylene glycol and glycerol. Figure 1 shows the impact of propylene glycol on the cavity radius, *R*, and *I*_3_ in PVA. It can be seen that *R* increases linearly with concentration, showing the increasing cavity radius upon plasticizer inclusion. There is a very modest decrease in the *I*_3_ parameter with propylene glycol concentration. This effect has been previously observed in plasticized films [13,33], and although in some cases has been attributed to a hole-filling mechanism [10], the increase in cavity radius with plasticizer concentration suggests that a likely reason for the decrease in *I*_3_ could be the result of quenching of *o*-Ps by the plasticizer or an increased number of sites where the positronium precursors can be trapped, rather than a decrease in void concentration. Although Equation (3) could be used to calculate the fractional free volume, the use of the determined positron parameters (τ_3_, *I*) to calculate free volume can only be justified when void concentration is the sole factor governing *o*-Ps intensity [32].

**Figure 2:**
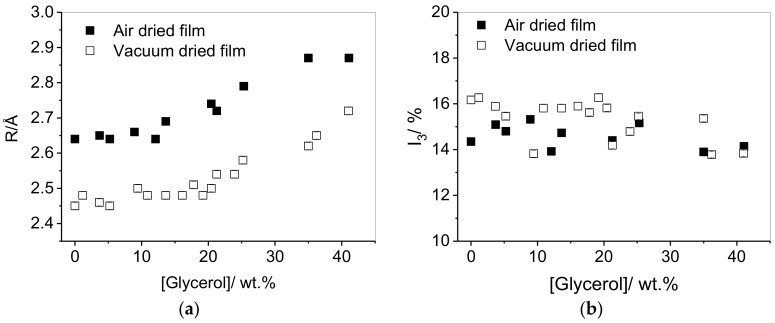
(**a**) Cavity radius in solution cast PVA (PVA-70-88) films as a function of glycerol concentration, (**b**) *o*-Ps intensity (*I*_3_) in solution cast PVA (PVA-70-88) films as a function of glycerol concentration. Data for air-dried films is represented by filled markers and data for vacuum dried films is represented by hollow markers.

**Figure 3:**
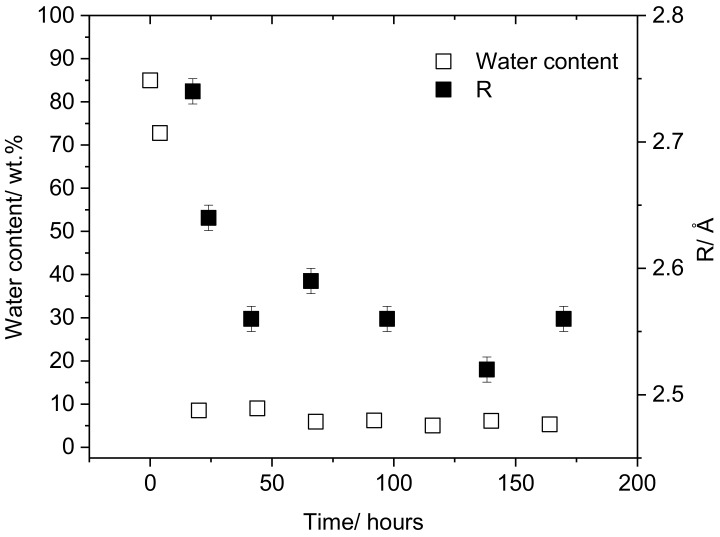
Decrease in non-plasticized PVA film water content over time during solution casting in air (hollow symbols) and decrease in cavity radius over time during the solution casting of films in air (filled symbols).

The presence of water and relative humidity significantly affects the mechanical properties of PVA and its blends [34]. To attempt to link this to its microscopic free volume properties, the impact of water inclusion on the plasticization of PVA by glycerol was therefore assessed by comparing air-dried and vacuum-dried films. Figure 2 shows the impact of glycerol on the free volume properties, and the effect of the presence of water in the film. The water content of the films after air-drying for 24 h (prior to drying under vacuum) was determined by TGA to be 8 wt % (Figure 3). As we found for propylene glycol, there is an increase in the size of the free volume holes with increasing glycerol content, confirming the plasticization effect. In contrast to propylene glycol, however, two regions are apparent for the glycerol system: the first at low concentrations, where increasing the plasticizer concentration has only a marginal increase on *R*, and the second at higher concentrations, where *R* increases significantly upon additional plasticizer inclusion. For both plasticizers, there was a slight decrease in the *I*_3_ parameter over the concentration range with increasing plasticization. Comparison of the PALS parameters in the vacuum-dried and air-dried films reveals that the systems follow identical trends, with the cavity radius systematically shifted to higher values when water is present, demonstrating the additional plasticization effect of water. The presence of water also causes a small, systematic shift to lower *I*_3_ values.

Figure 3 shows the decrease in water content over an 8-day drying period during solution casting in the absence of additional plasticizers, from 8.5% at 24 h, equilibrating at approximately 5–6% after 3 days. This is accompanied by a decrease in the cavity radius, corresponding to the decreasing void size as water is lost from the film. The presence of low amounts of water has a significant impact on the cavity radius. This 3.2% decrease in water content leads to a 4% reduction in *R*.

**Figure 4:**
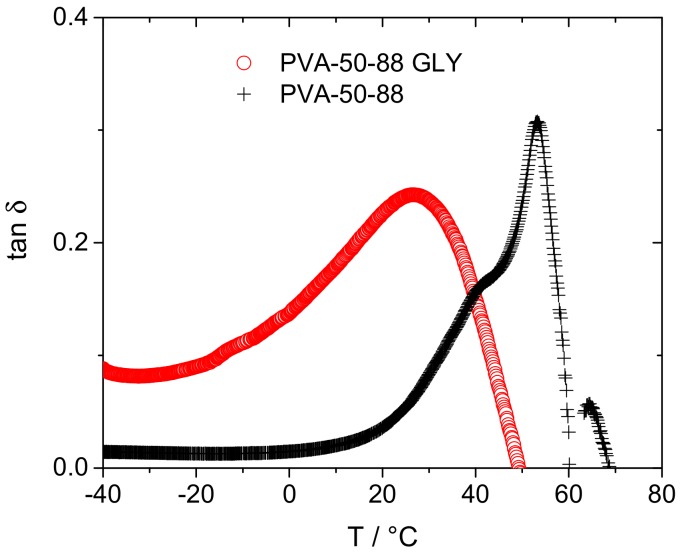
DMA data for pure air-dried PVA-50-88 (black) and air-dried glycerol-plasticized PVA-50-88 (red) following equilibration in air at 33% relative humidity.

**Figure 5:**
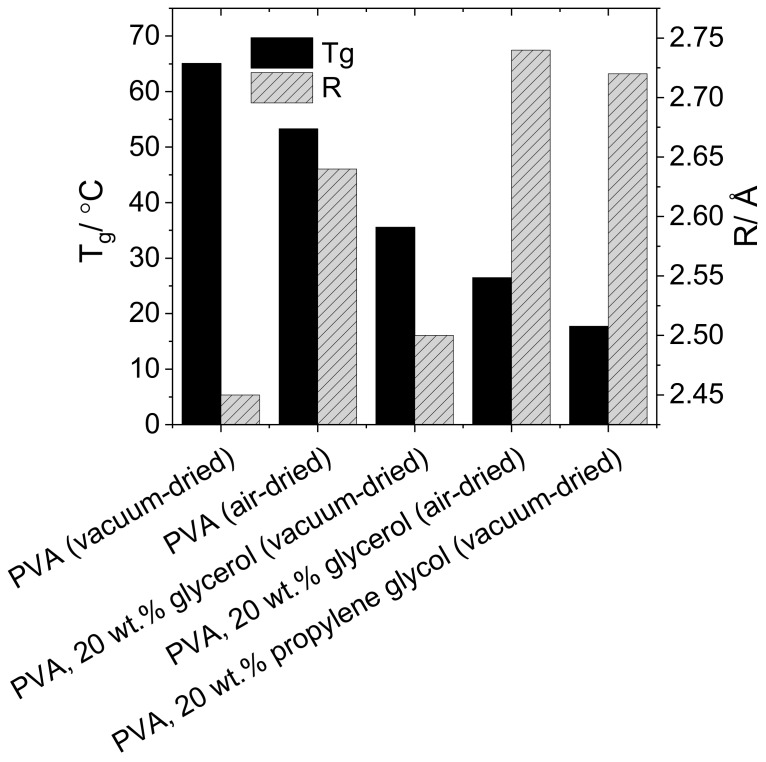
Glass transition temperatures and cavity radii for solution cast PVA resins with both in the presence and absence of plasticizers.

It would be expected that an increase in free volume cavity radius would lead to a decrease in the *T*_g_. Although not easily calorimetrically detectable, the glass transition temperatures of the solution cast resins can be determined using DMA (Figure 4), allowing the effect of plasticizer inclusion on the macroscopic polymer properties to be compared with the microscopic free volume properties. The *T*_g_ and cavity radii of partially-hydrolyzed PVA, both non-plasticized and in the presence of 20 wt % glycerol and 20 wt % propylene glycol, are compared Table 2 and Figure 5. Propylene glycol causes a greater depression in the glass transition than glycerol, 47.4 °C vs. 29.6 °C (14% vs. 9% reduction in *T*_g_ in K), correlating well to the greater % increase in cavity radius upon propylene glycol compared to glycerol incorporation (11% vs. 2%), although the magnitudes of the variation in these parameters differ greatly.

**Figure 6:**
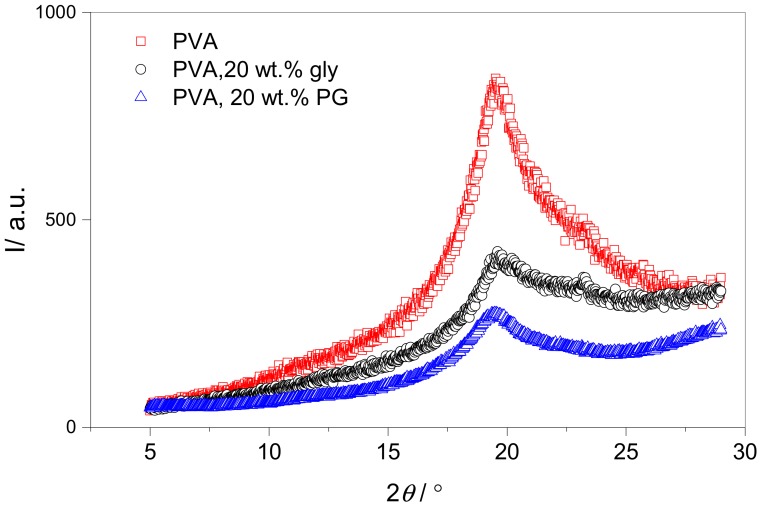
XRD patterns for vacuum dried, solution cast film of pure PVA-50-88 (red), with 20 wt % glycerol incorporated (black) and with 20 wt % propylene glycol incorporated (blue).

To gain further insight into the nature of the resins and the effect of plasticizer incorporation, XRD was used to assess the crystallinity of the samples, as shown in Figure 6. Although PALS measures the free volume void size in the amorphous regions in the polymer, insight into these different plasticizing behaviors can be gained from the polymer crystal structure. The characteristic peak at approximately 20° confirms the semi-crystalline nature of the polymer [35]. The apparent crystallite size in the PVA samples, was estimated from the inverse peak width using Scherrer equation,

(4) $$t_{crys}=\frac{k\lambda }{\beta \cos\theta }$$

where *k* is a shape factor for the crystallite, here assumed to be unity, *λ* is the X-ray wavelength, *β* is the FWHM peak width determined after linear background subtraction, and *θ* is the scattering angle of the peak. Crystallite sizes estimated this way were approximately 2.5 nm, and are listed in Table 3. We note that this is a lower bound to the crystallite size based on the assumption that this is the only contribution to the measured XRD peak widths. The main effect for both plasticizers is to reduce the magnitude of the peak when compared to the background scattering. It is difficult to quantify this reduction in crystallinity accurately, because the crystalline peaks are insufficiently sharp to definitively resolve from the background X-ray scattering arising from the amorphous PVA. For completeness, we include the integrated peak to background ratio in Table 3. It can clearly be seen that the incorporation of 20% plasticizer significantly reduces the degree of crystallinity, and our results suggest that this reduction may be greater for propylene glycol than for glycerol.

**Figure 7:**
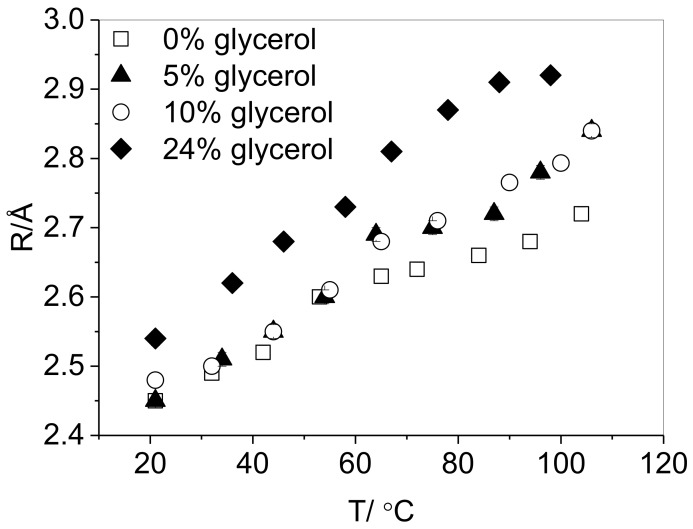
Influence of temperature on the *o*-Ps lifetime in non-plasticized PVA and PVA containing 0, 5, 10 and 24 wt % glycerol. Error bars are within the size of the data points.

The effect of temperature on the positron lifetimes in glycerol-plasticized PVA was investigated up to 110 °C. Figure 7 shows the temperature dependence of τ_3_ for three glycerol loadings. At each loading, an increase in temperature results in longer *o*-Ps lifetimes, corresponding to an increase in cavity radius. A small step increase in *R* is apparent in the region 45–65 °C for PVA containing 0, 5 and 10 wt % glycerol. This is likely to be due to the glass transition [21], which is consistent with the *T*_g_ determined by DMA for the unplasticized resin. The absence of such a transition observed in the resin with a 24% glycerol loading is consistent with the much lower *T*_g_ of highly plasticized PVA (27 °C, Table 2). The behavior of the τ_3_ parameter confirms the negligible change in a cavity radius of up to 10 wt % glycerol relative to that of pure PVA-70-88. Up to 65 °C, this level of glycerol incorporation has no effect on *R*; throughout the entire temperature range studied, the films containing 5 and 10% glycerol are shown to contain free voids of the same size. A divergence at higher temperatures is, however apparent, although films containing 5% and 10% glycerol appear to contain voids of the same radius throughout the entire temperature range studied.

### 3.2. Influence of the Degree of Hydrolysis on PVA Free Volume Properties

**Figure 8:**
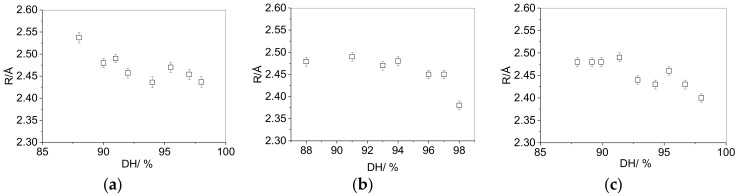
Effect of degree of hydrolysis on the cavity radius in non-plasticized, vacuum-dried PVA resins of different molecular weights: (**a**) PVA-23-(DH), (**b**) PVA-50-(DH), (**c**) PVA-130-(DH).

The degree of hydrolysis can affect both the nature of the interactions between polymer chains and the interactions between the polymer and small additive molecules such as plasticizers. PALS measurements were obtained for PVA films of DH ranging from 88% to 98% for three molecular weights of PVA (13,000–23,000, 31,000–50,000 and 125,000–130,000 g·mol^−1^). Figure 8 shows no significant change in the *o*-Ps lifetime with molecular weight, confirming that the chain ends do not measurably contribute to the plasticization of the PVA in this molecular weight range. For each molecular weight, a modest decrease in *R* is observed when DH is increased from 88% to 98%, suggesting that the replacement of a higher fraction of acetate groups with hydroxyl groups leads to a reduction in the void size in the PVA resin.

**Figure 9:**
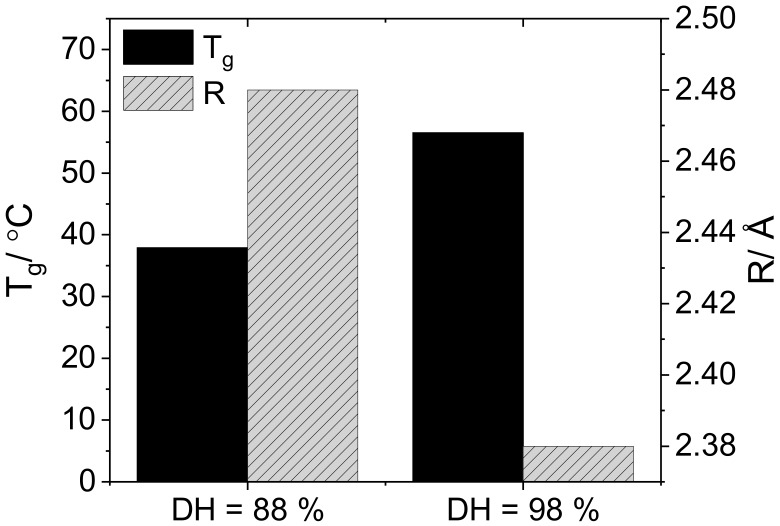
Change in cavity radius and *T*_g_ of vacuum-dried PVA films upon increasing the degree of hydrolysis from 88% to 98%.

The link between the macroscopic properties of the polymer and the microscopic free volume properties can be assessed by comparing the glass transitions to the cavity radii (Figure 9). Table 4 shows the effect of the degree of hydrolysis on the glass transition of air-dried films (*M*_w_ = 31–50 kg·mol^−1^). Again, the decrease in cavity radius upon increasing the degree of hydrolysis is also reflected in the increase in glass transition temperature.

**Figure 10:**
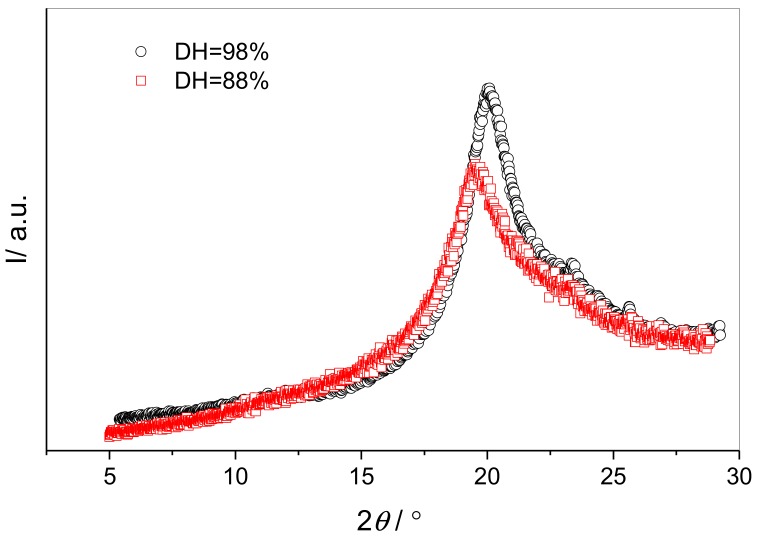
Superimposed XRD patterns for vacuum-dried PVA resins with degrees of hydrolysis of 88% (red) and 98% (black).

The superimposed XRD patterns for PVA resins of degrees of hydrolysis of 88% and 98% (Figure 10) confirm that altering the degree of hydrolysis of the resin in this range does not greatly alter the characteristic interchain spacing of crystal structure. It is however apparent that the PVA-50-98, having a higher degree of hydrolysis, also has a higher degree of crystallinity, as evidenced by the taller, Bragg peak, and greater peak integral. Although PALS measures only voids present in the amorphous region of the polymer, and crystallinity should therefore not directly affect cavity radius, the simultaneous increase in crystallinity and reduction in void size demonstrates the profound effect of this small increase in the degree of hydrolysis of the resin, with significant implications for additive interactions with the polymer.

**Figure 11:**
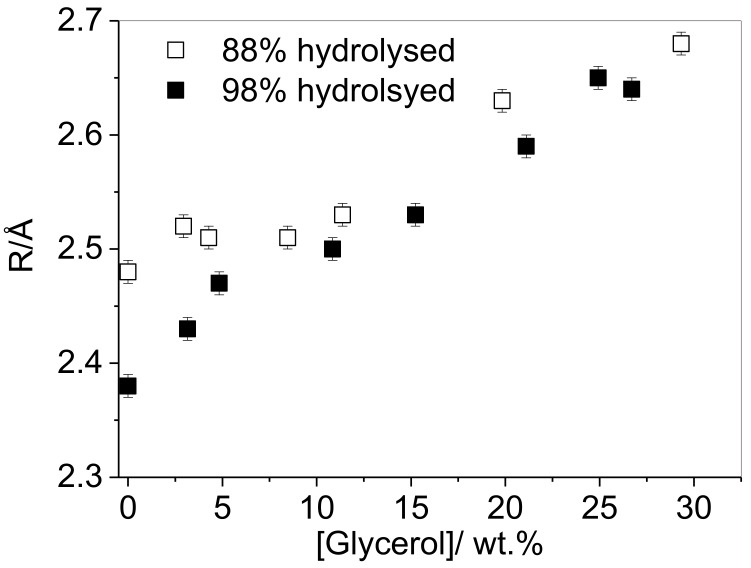
Comparison of the effect of glycerol loading on the cavity radius in solution cast, vacuum-dried PVA resins of different degrees of hydrolysis (open symbols: PVA-50-88, filled symbols PVA-50-98).

The effect of DH on interactions with additives, and the resulting impact on free volume properties, were assessed by comparing the behavior of the *o*-Ps lifetime in glycerol-plasticized films of 88% and 98% hydrolyzed PVA resins (*M*_w_ = 31–50 kg·mol^−1^). Figure 11 shows that the behavior of PVA-50-88 is consistent with the plasticization of PVA-70-88 (Figure 2); up to a critical concentration glycerol incorporation causes negligible change in cavity radius, but as the loading is increased beyond this point, this parameter increases significantly. In contrast, a linear increase in cavity radius with glycerol loading is observed throughout the entire plasticizer concentration range for PVA-50-98.

## 4. Discussion

### 4.1. Effect of Plasticization on the Free Volume Properties of PVA

A modest decrease in *I*_3_ is apparent upon incorporation of both glycerol and propylene glycol to the PVA resin. Although this parameter is related to the void concentration, an increase in plasticizer concentration is not expected to reduce the number of free volume cavities. An explanation for the decrease in this parameter was, however, proposed by Mohamed et al. [27] who observed a decrease in the *I*_3_ of PVA with increasing temperature. They suggested that this was a result of an increased number of sites where the positronium precursors can be trapped, although little is known about the nature of these sites. Alternatively, the slight decrease in *I*_3_ upon glycerol and propylene glycol incorporation could be attributed to the possible quenching of *o*-Ps by the plasticizer.

The variation of *o*-Ps lifetime with plasticizer concentration in partially hydrolyzed (87–90% DH) PVA is different for the two plasticizers studied. Although a linear increase in cavity radius was found throughout the whole propylene glycol concentration range, up to 20% glycerol could be incorporated into the same resin, whilst having negligible impact on the void size. One interpretation of this result is phase separation of the glycerol from matrix, and the formation of pure plasticizer domains after a critical loading. However, we discount the possibility of phase separation in this case because it would result in another location for *o*-Ps annihilation, and in a poor fit of the PALS data with a three-component model. Above 20 wt % plasticizer, however, the PALS data could still be fitted well with three components, with negligible improvement in variance attained by introducing a fourth, making this explanation unlikely. Additionally, it has been shown that glycerol and partially-hydrolyzed (87–89%) PVA are compatible to 39 wt %, determined by the plasticizer loading at which the heat of fusion drastically reduces [16].

An explanation for the change in the cavity radius with plasticizer incorporation behavior can be postulated by considering the nature of the interactions of the plasticizers with the polymer. It is widely accepted that the plasticization of PVA can be attributed to the formation of hydrogen bonds between plasticizer and polymer chains [6,7]. As glycerol has a higher –OH group functionality than propylene glycol, it could therefore be suggested that glycerol more readily forms hydrogen bonds with the hydroxyl groups of PVA chains, whilst causing a minimal impact on chain separation up to a certain loading. The methyl group of propylene glycol, however, is likely to cause more disruption in the inter-chain hydrogen bonding, and may inhibit plasticizer-polymer hydrogen bonding, leading to an immediate increase in cavity radius. This can also be considered in terms of the macroscopic polymers, notably, the further reduction in crystallinity of the propylene glycol plasticized PVA when compared to the same material plasticized with glycerol (Figure 6). Furthermore, as the PALS measurements were performed at 20 °C, the unplasticized polymer and polymer containing 20 wt % glycerol will be below its glass transition, while the film containing 20 wt % propylene glycol will be above *T*_g_ (Table 2). As Figure 7 shows that the glass transition causes only slight changes in the free volume cavity radii, it is therefore sensible to suggest that the substantial increase in hole radius as a result of the nature of the PVA-propylene glycol interactions results in the significant depression of the observed *T*_g_. A lesser depression in the *T*_g_ of the glycerol-plasticised films is measured, which remains above the temperature at which PALS measurements were conducted, corresponding well to the slight increase in cavity radius detected.

The significant plasticizing behavior of water should also be considered, not least since water or water vapor is ubiquitous in virtually all industrial applications for PVA. A comparison of Figure 1, Figure 2 and Figure 3 shows that the inclusion of 8 wt % water has a greater impact on cavity radius than either of the two plasticizers at this loading. A two-fold mechanism of plasticization of PVA by water has been previously suggested from studies of the properties of PVA as water is absorbed. As well as increasing the size of the free volume cavities, a lubrication effect was identified, which promotes chain mobility and disrupts hydrogen bonding [36]. For the properties of solution-cast films, the reverse process is more relevant; therefore, in the present work, the change in PALS parameters during the drying of the film was studied. Here, we focus on the relatively low water content and partially-hydrolyzed PVA relevant to solution cast film, as opposed to the very broad range studied by Hodge et al. [36]. Nevertheless some similar behavior is was found. In the range between 8% and 30% water, an increase in cavity radius and large decrease in *I*_3_ (from 27% to 20.5%) with water content was reported, consistent with that observed in the present study with the reverse (drying) system. A similar argument could be applied, addressing the modest decrease in *I*_3_ upon plasticizer incorporation. However, from the increasing number of free volume cavities as the water content in the film decreases, it could also be postulated that, as well as increasing the size of the holes, water is also occupying a fraction of the free volume cavities, decreasing their relative concentration. This contrasts the behavior observed for glycerol and propylene glycol, where only a modest decrease in *I*_3_ is apparent as glycerol loading increases. It is also possible that a decrease in *o*-Ps intensity could be attributed to dynamic reordering of the PVA chains upon water loss. Thimmegowda et al. [37] have observed an initial decrease in *I*_3_ with water sorption in poly(2-hydroxyethyl methacrylate), which was attributed to the reordering of the polymer chains as a result of the swelling by water. It is therefore feasible that the reverse argument can be applied here; a reordering of PVA chains upon water loss could result in the division of comparatively large free volume holes into smaller ones (with a decrease in size greater than can be accounted for by water loss from the cavities alone), thus increasing the number of free volume holes. Despite the apparent decrease in *I*_3_ with increasing water content, indicating the decrease in concentration of free volume cavities, the high frequency of the plasticizer motion means that the water occupying the cavities can still contribute to polymer chain mobility.

By comparing the change in *τ*_3_ with glycerol concentration for air-dried and vacuum-dried films (Figure 2a), it is apparent that water has a more significant effect on the void volume. This can be attributed to the greater ability of water to disrupt the hydrogen bonding network within the PVA resin compared to glycerol. The observation of the significant plasticization effect by water in films already containing other plasticizers highlights the industrial significance of humidity, and its impact on PVA matrix properties.

### 4.2. Influence of the Degree of Hydrolysis on PVA Free Volume Properties

The decrease in *τ*_3_ with DH from 88–98% for three molecular weights of PVA shows how the replacement of residual acetate groups by hydroxyl groups causes a decrease in void size in the matrix, with similar behavior observed for each molecular weight of PVA. This is likely to be a result of an increased number density of hydrogen bonding sites and the formation of a tighter structure. The ratio of vinyl acetate to vinyl alcohol has previously been varied from 20% vinyl alcohol groups to 85% vinyl alcohol groups [19]. At low levels of hydrogen bonding, increasing the vinyl alcohol content led to an increase in free volume, suggesting the formation of a more open structure. After a critical value, however, (35% vinyl alcohol) a sudden decrease in the free volume of the system was observed over a very narrow range. This was suggested to be a result of an increased number of hydrogen bonding sites, as well as the replacement of the carbonyl group hydrogen bond acceptors which favor a more open structure than hydroxyl groups. In the present work, where the vinyl group content is higher (88%), the films are well into the post-network collapse region. As the hydrogen bond length lies in a narrow range of 2.6–3.1 Å, there are stringent steric restrictions in the network when a high proportion of hydroxyl group acceptors are present. It could therefore be expected that following network collapse, where a critical concentration of hydrogen bond donors and hydroxyl group acceptors is needed to establish the formation of the tighter network, the structure would be influenced very little by a further increase in the concentration of hydroxyl groups. Despite this, the slight decrease in cavity radius between 88% and 98% hydrolysis suggests that that the disappearance of nearly all acetate groups permits the formation of an even tighter network. The size of the microscopic free volume is reflected in the macroscopic polymer properties, as the glass transition temperature is shown to increase as void size decreases upon formation of a tighter network.

This result could have significant implications for the interactions of other additives in the PVA matrix, and significantly, for the plasticization of the resin, particularly as it has been previously demonstrated that certain surfactants have a significant affinity for glycerol, but much less for PVA [8,9]. By comparing the plasticization effect of glycerol in partially- and fully-hydrolyzed resins it can be seen that although a limited content of glycerol can be incorporated into the partially-hydrolyzed resin without affecting sizes of voids present, the inclusion of any glycerol into an almost fully-hydrolyzed resin also increases the cavity radius (Figure 11). This suggests that in the partially-hydrolyzed resin, the glycerol molecules interact preferentially with the residual acetate groups, or occupy the larger cavities formed by the presence of acetate groups. In the absence of acetate groups which disrupt the inter-chain hydrogen bonding, introduction of plasticizer will disrupt the tighter hydrogen bonded network, even at very low loadings.

## 5. Conclusions

PALS has revealed interesting differences in the plasticization behaviors of propylene glycol, glycerol, and water, which is attributed to their different functionality, and thus the different extent of interactions between the matrix and additive. It was noted that water has a more significant effect of free volume cavities than either glycerol or propylene glycol. Increasing the DH of the PVA resin causes a decrease in free volume cavity radius, indicating that the absence of acetate groups permits the formation of a tighter network. When considering both the DH of the resin and the incorporation of plasticizers, the size of the microscopic free volume is shown to be reflected in the glass transition temperature; however, the relationship between these parameters is complex. From the different plasticization behavior of glycerol in partially- and fully-hydrolyzed PVA, we propose that glycerol preferentially interacts with PVA in the vicinity of residual acetate groups which permit a limited concentration of the plasticizer to be accommodated in PVA without increasing the size of existing voids present in the matrix. The insights and information gained from PALS show its utility as a technique for exploring the interaction between the polymer and plasticizer interactions, and the relationship between molecular structure and bulk material properties.

## Figures and Tables

**Table 1:** Details of PVA resins used for positron annihilation studies.

Material Reference	*M*_w_/kg · mol^−1^	DH/%
PVA-70-88	30–70	87–90
PVA-23-98	13–23	98
PVA-23-88	13–23	87–89
PVA-50-98	31–50	98–99
PVA-50-88	31–50	87–89
PVA-125-98	125	98
PVA-130-88	130	88

**Table 2:** Comparison of the effect of plasticization and residual water on the glass transition and cavity radius of solution cast PVA films.

Plasticiser	Drying Method	R/Å	*T*_g_/°C
None	Air	2.64	53.3
None	Vacuum	2.45	65.1
Glycerol	Air	2.74	26.8
Glycerol	Vacuum	2.50	35.5
Propylene glycol	Vacuum	2.72	17.7

**Table 3:** Comparison of the effect of plasticization and residual water on the glass transition and cavity radius of solution cast PVA films. Integrals and FWHM of XRD peaks for vacuum-dried films containing non-plasticized PVA, PVA plasticized with glycerol and PVA plasticized with propylene glycol.

Sample	I_peak_/I_peak_ + I_baseline_	FWHM/°	t_crys_/nm
PVA	0.42	3.8	2.4
PVA/glycerol	0.12	4.0	2.2
PVA/propylene glycol	0.10	2.9	3.1

**Table 4:** Comparison of the cavity radius and *T*_g_ in vacuum-dried films with degrees of hydrolysis PVA-50-88 and PVA-50-98.

DH/%	R/Å	*T*_g_/°C
88	2.64	53.3
98	2.45	65.1
